# Index Catalogue of the Library of the Surgeon General's Office, United States Army—Authors and Subjects

**Published:** 1886-01

**Authors:** William Beer


### Index Catalogue of the Library of the Surgeon General's Office, United States Army—Authors and Subjects.

| | | | | | |
|---|---|---|---|---|---|
| Vol. I. | pp. v | 126- 886. | 4° | Washington | 1880 |
| Vol. II. | pp. | 12- 990. | " | " | 1881 |
| Vol. III. | pp. | 11-1020. | " | " | 1882 |
| Vol. IV. | pp. | 12-1033. | " | " | 1883 |
| Vol. V. | pp. | 11-1055. | " | " | 1884 |
| Vol. VI. | pp. | 11-1051. | " | " | 1885 |

(Each volume contains a list of periodicals indexed to date of publication.)

Medical bibliography has never been so manificently treated as in the volumes before us. The preface warns the reader that this splendid work is only the catalogue of a special collection, but it is probably the best collection in the world.

Those who have studied the matter will be aware of the necessity of such assistance as this catalogue can give, for all existing publications fall short of the requirements of the investigator of special subjects in medical literature.

Some of the medical bibliographies are mere fragments, such as that of Atkinson, of York, which is intensely amusing, but stops short at the letter C; others, as that of Ploncquet, are full of error; and others again by an over refinement of classification, such as that of the almost omniscient Thomas Young, are comparatively useless to the student.

Of all methods of cataloguing, that known as the index catalogue is undoubtedly the most serviceable to the general public. It is, however, necessarily bulky, for each book appears first under the author's name, secondly under its own title, and thirdly classified with others upon the same subject.

The first of the two sets of numerals in the description of the volumes give the numbers of the pages containing lists and addenda of the medical journals indexed, for under each subject heading are given the articles in journals relating to it.

When we state that 27 pages are devoted to works on the abdomen, 34 to amputation, 39 to anatomy, 65 to aneurisms, and 42 to arteries, some idea can be formed of the richness of the collection, the thoroughness of the cataloguing, and of the relative importance of these subjects in medical literature.

The heading, Bibliography, covers no less than 12 pages, and gives certainly the most complete list of such works ever published. The description of a few of the books named might have been amplified with advantage. The preservation of catalogues of private libraries and of booksellers is especially to be commended, for even when, as is too often the case, they are compiled in a loose and slovenly way, they are matter of inestimable value to the bibliographer.

The literary student of dental surgery will see with pleasure the number of editions of Aetius, Albucasis, Avicenna, Celsus, and Galen, the earliest writers on the teeth, possessed by the National Medical Library, but his especial interest will lie in pages 681 to 691 of volume 3, which include the books on dental and oral surgery.

On this subject, however, the collection falls short of the general standard of completeness, amongst other notable desiderata being the great works of Carabelli—Systematisches Handbuch der Zahnheilkunde, 2v, mit Atlas in 4°, Wien, 1831–44.

Cuvier, Des dents des mammiferes considerees comme caracteres zoologiques, 8v, Paris, 1825.

The very complete list of articles on dental subjects which have appeared in periodicals devoted to general medicine is likely to be of great value.

The summary given at the commencement of the 6th volume shows the progress of this census of the literary wealth of the nation, medically considered, to the date of issue.

Catalogued volumes, 33,265.

Pamphlets, 47,325; journal articles, 219,154.

Portraits, 4,335.

In conclusion we would draw the attention of the members of our own and of the medical profession to the necessity of the assistance of each and all in the completion of this national collection. The scattered pamphlets which lie about in most houses uncatalogued and therefore unconsulted become of great value in a well-ordered library, and we are certain that Dr. Billings, the Librarian of the Surgeon General's Library, to whose initiative and perseverance is due the appearance of this catalogue, will be obliged to any of them who will send him lists of their duplicates or unneeded volumes. WILLIAM BEER.

